# Hypoxia-Mediated ATF4 Induction Promotes Survival in Detached Conditions in Metastatic Murine Mammary Cancer Cells

**DOI:** 10.3389/fonc.2022.767479

**Published:** 2022-06-30

**Authors:** Violet A. Kiesel, Madeline P. Sheeley, Emily M. Hicks, Chaylen Andolino, Shawn S. Donkin, Michael K. Wendt, Stephen D. Hursting, Dorothy Teegarden

**Affiliations:** ^1^ Purdue University, Department of Nutrition Science, West Lafayette, IN, United States; ^2^ Purdue University, Department of Animal Science, West Lafayette, IN, United States; ^3^ Purdue University, Department of Medicinal Chemistry and Molecular Pharmacology, West Lafayette, IN, United States; ^4^ Purdue University, Purdue University Center for Cancer Research, West Lafayette, IN, United States; ^5^ University of North Carolina at Chapel Hill, Department of Nutrition, Chapel Hill, NC, United States; ^6^ University of North Carolina at Chapel Hill, Lineberger Comprehensive Cancer Center, Chapel Hill, NC, United States; ^7^ University of North Carolina at Chapel Hill, Nutrition Research Institute, Kannapolis, NC, United States

**Keywords:** breast cancer, metastasis, hypoxia, integrated stress response (ISR), cell stress, ATF4 activating transcription factor 4

## Abstract

Regions of hypoxia are common in solid tumors and drive changes in gene expression that increase risk of cancer metastasis. Tumor cells must respond to the stress of hypoxia by activating genes to modify cell metabolism and antioxidant response to improve survival. The goal of the current study was to determine the effect of hypoxia on cell metabolism and markers of oxidative stress in metastatic (metM-Wnt^lung^) compared with nonmetastatic (M-Wnt) murine mammary cancer cell lines. We show that hypoxia induced a greater suppression of glutamine to glutamate conversion in metastatic cells (13% in metastatic cells compared to 7% in nonmetastatic cells). We also show that hypoxia increased expression of genes involved in antioxidant response in metastatic compared to nonmetastatic cells, including glutamate cysteine ligase catalytic and modifier subunits and malic enzyme 1. Interestingly, hypoxia increased the mRNA level of the transaminase glutamic pyruvic transaminase 2 (Gpt2, 7.7-fold) only in metM-Wnt^lung^ cells. The change in Gpt2 expression was accompanied by transcriptional (4.2-fold) and translational (6.5-fold) induction of the integrated stress response effector protein activating transcription factor 4 (ATF4). Genetic depletion ATF4 demonstrated importance of this molecule for survival of hypoxic metastatic cells in detached conditions. These findings indicate that more aggressive, metastatic cancer cells utilize hypoxia for metabolic reprogramming and induction of antioxidant defense, including activation of ATF4, for survival in detached conditions.

## Introduction

Regions of hypoxia, or low oxygen tension, are prevalent in solid tumors and are inversely associated with survival in patients with cancer (1, 2). Hypoxia is a source of cell stress, as reduced availability of molecular oxygen increases oxidative stress through production of mitochondrial reactive oxygen species (ROS) and endoplasmic reticulum stress through impaired folding of nascent peptides (3–5). Cancer cells respond to hypoxia-induced cell stress through multiple overlapping mechanisms, including hypoxia signaling *via* the transcription factor hypoxia-inducible factor 1α and activation of the integrated stress response (ISR) (6–9). Hypoxia signaling and activation of the ISR, *via* increased translation of its main effector protein activating transcription factor 4 (ATF4), can induce expression of genes involved in cell proliferation and apoptosis, metabolism, and antioxidant defense (6, 7, 9).

In hypoxia, nutrient metabolism is reprogrammed to limit entry and oxidation of metabolites in the tricarboxylic acid (TCA) cycle, consequently limiting production of ROS at the electron transport chain (10–14). Hypoxia, for example, reduces flux of pyruvate into the TCA cycle by upregulating pyruvate dehydrogenase kinase 1 (PDK1) and lactate dehydrogenase A (LDHA), collectively promoting conversion of pyruvate to lactate (10, 11). Similarly, hypoxia reduces flux of glutamine into the TCA cycle as α-ketoglutarate (αKG), and promotes reductive carboxylation of αKG to citrate, which may be used for fatty acid synthesis (13). Shunting metabolites away from oxidation in the TCA cycle limits production of the coenzymes NADH and FADH_2_, thus limiting activity at the electron transport chain and reducing oxidative stress. In addition, hypoxia signaling activates genes required for production of the antioxidant molecules NADPH and glutathione, thereby alleviating oxidative stress by increased clearance of ROS in hypoxia (7). Thus, metabolic reprogramming represents an important axis of reducing oxidative stress in hypoxia.

Hypoxia also activates the ISR pathway. In this pathway, kinases are activated by specific cell stresses, including endoplasmic reticulum stress, oxidative stress, and hypoxic stress (9). The activated kinases phosphorylate elongation initiation factor 2α, which globally reduces mRNA translation while simultaneously increasing translation of select transcripts, including *ATF4* (9, 15). ATF4 is the main effector protein of the ISR, and it modifies gene expression to reduce cell stress and restore cell homeostasis, or induce apoptosis if the degree of cell stress is too high to be resolved (9). Mechanisms by which ATF4 reduces cell stress include altering cell metabolism and upregulating oxidative stress response (16, 17). Given its integral role in reducing cell stress, previous work has identified ATF4 as key protein involved in metastatic processes such as migration (18, 19) and resistance to anoikis (20), suggesting that its expression is not only critical to cell survival, but also to cancer progression.

Although previous work has examined the role of ATF4 in cancer cell metabolism (21), prometastatic outcomes (18–20), and tumor growth *in vivo* (20, 22–26), currently there is a gap in research examining ATF4 induction in response to hypoxia throughout the stages of cancer progression. In the current study we employed nonmetastatic M-Wnt and metastatic metM-Wnt^lung^ murine mammary cancer cell lines to address this research gap. metM-Wnt^lung^ cells have elevated expression of genes involved in stress response compared to nonmetastatic M-Wnt cells, suggesting this isogenic series of cell lines may serve as a good cell model of stress accrual that occurs throughout the course of metastatic progression (27). In the present work, the effects of hypoxia on metabolic adaptation and processes involved in metastatic progression in M-Wnt and metM-Wnt^lung^ cells were assessed. We hypothesized that ATF4 contributes to survival of hypoxic metM-Wnt^lung^ cells in detached conditions.

## Materials and Methods

### Cell Culture

Murine M-Wnt and metM-Wnt^lung^ mammary cancer cells were cultured in Dulbecco’s Modified Eagle’s Medium (DMEM, Sigma, St. Louis, MO) with 5 mM glucose, 2 mM glutamine, no sodium pyruvate, with 10% final concentration fetal bovine serum (Gibco, Waltham, MA) and 1% final concentration penicillin-streptomycin (Gibco). M-Wnt cells were derived from the primary tumor of a mouse mammary tumor virus Wnt-1 transgenic mouse, and do not metastasize to the lung or liver when orthotopically injected into syngeneic C57BL/6 mice, and therefore serve as a model of nonmetastatic mammary cancer (27, 28). metM-Wnt^lung^ cells were derived from a lung metastatic lesion which formed after serially passaging M-Wnt cells through five generations of non-obese diabetic severe combined immunodeficient mice, and preferentially metastasize to the lung when orthotopically injected into C57BL/6 mice (27). For experiments in hypoxia, cells were seeded and attached under normoxic conditions (~20% O_2_, 5% CO_2_, ~75% N_2_). The following day, media was changed and cells were transferred to a Billups-Rothenberg modular incubator chamber (Billups Rothenberg, Del Mar, CA). The modular incubator chamber was flushed with at least 100 L of hypoxic gas mixture (1% O_2_, 5% CO_2_, 94% N_2_), and cells were incubated at 37°C.

### Viability Assay

Cells were seeded into 96 well plates, attached in normoxia overnight, and subsequently cultured in normoxia or hypoxia for 48 h. Following incubation, media was removed and replaced with 1X (0.5 mg/mL) MTT in serum-free media. Plates were incubated for two hours at 37°C, crystals were dissolved in dimethyl sulfoxide (DMSO), and absorbance was determined at 570 nm with a Biotech spectrophotometer.

### RNA Isolation and qRT-PCR

RNA was isolated using TRI-Reagent (Molecular Research Center, Cincinnati, OH) according to the manufacturer’s protocol. RNA was reverse transcribed using MMLV reverse transcriptase (Promega, Madison, WI). Real-time quantitative PCR was performed using LightCyler 480 SYBR Green I Master Mix (Roche, Indianapolis, IN). The comparative Ct method (2^-ΔCt^) was used to normalize mRNA data to the indicated reference group, using *18s* as a housekeeping gene. Primers for qRT-PCR are listed in **Table 1**.

**Table 1:** Primers used for qRT-PCR.

*Atf4*	Forward: 5’-CCTGAACAGCGAAGTGTTGG-3’ Reverse: 5’-TGGAGAACCCATGAGGTTTCAA-3’
*Gclc*	Forward: 5’-GGGGTGACGAGGTGGAGTA-3’ Reverse: 5’-GTTGGGGTTTGTCCTCTCCC-3’
*Gclm*	Forward: 5’-AGGAGCTTCGGGACTGTATCC-3’ Reverse: 5’-GGGACATGGTGCATTCCAAAA-3’
*Glud1*	Forward: 5’-CCCAACTTCTTCAAGATGGTGG-3’ Reverse: 5’-AGAGGCTCAACACATGGTTGC-3’
*Got2*	Forward: 5’-GGACCTCCAGATCCCATCCT-3’ Reverse: 5’-GGTTTTCCGTTATCATCCCGGTA-3’
*Gpt2*	Forward: 5’-AACCATTCACTGAGGTAATCCGA -3’ Reverse: 5’-GGGCTGTTTAGTAGGTTTGGGTA -3’
*Gss*	Forward: 5’-CAAAGCAGGCCATAGACAGGG-3’ Reverse: 5’-AAAAGCGTGAATGGGGCATAC-3’
*Ldha*	Forward: 5’-AAACCGAGTAATTGGAAGTGGTTG-3’ Reverse: 5’-TCTGGGTTAAGAGACTTCAGGGAG-3’
*Me1*	Forward: 5’-TCAACAAGGACTTGGCTTTTACT-3’ Reverse: 5’-TGCAGGTCCATTAACAGGAGAT-3’
*Pdk1*	Forward: 5’-ACAAGGAGAGCTTCGGGGTGGATC-3’ Reverse: 5’-CCACGTCGCAGTTTGGATTTATGC-3’
*Psat1*	Forward: 5’-CAGTGGAGCGCCAGAATAGAA-3’ Reverse: 5’-CCTGTGCCCCTTCAAGGAG-3’
*18s*	Forward: 5’-ATCCCTGAGAAGTTCCAGCA-3’ Reverse: 5’-CCTCTTGGTGAGGTCGATGT-3’

### Western Blotting

Cell samples were washed twice with ice-cold phosphate-buffered saline (PBS) prior to harvesting in radioimmunoprecipitation assay (RIPA) buffer (Cell Signaling, Danvers, MA) with 1% phenylmethanesulfonyl fluoride protease inhibitor (PMSF, Cell Signaling) and phosphatase inhibitor cocktail 2 (P5726, Sigma). Cells were lysed with sonication and vortexing, and cell debris was pelleted with centrifugation at 14,000 RPM for 15 minutes. Protein concentration of the supernatant was determined with Pierce bicinchoninic acid assay (BCA) protein assay kit (ThermoFisher, Waltham, MA). Equal amounts of protein (25 μg) were separated on polyacrylamide gels and transferred to 0.2 μm nitrocellulose membranes (Bio-Rad, Hercules CA). Membranes were probed with antibodies for actin, ATF4, and GPT2 (Actin Cell Signaling #4970, 1:4000; ATF4 Cell Signaling #11815, 1:1000; GPT2 ThermoFisher #16757-1-AP, 1:1000) overnight in 5% nonfat dry milk. Membranes were incubated with secondary antibodies (LiCor # 926-68073, 1:10,000) for one hour at room temperature. Protein was detected using an Odyssey CLx imaging system (Li-Cor, Lincoln, NE).

### Low Attachment Survival Assay

Cells were incubated for 48 h in normoxia or hypoxia. Cells were then removed from the modular incubator chamber, trypsinized, and 50 μL of cell suspension was rapidly re-seeded at equal cell densities into polyhydroxyethylmethacrylate (poly-HEMA) coated plates. To assess viability, 5 μL of 10X (5 mg/mL) MTT solution (Sigma) was added to each well and the plate was incubated at 37°C for two hours. MTT crystals were dissolved in 150 μL DMSO and absorbance was read at 570 nm utilizing a Biotech spectrophotometer.

### Transwell Migration Assay

Cells were incubated in normoxia or hypoxia for 48 h. Following incubation, cells were removed from the modulator incubator chamber, trypsinized, pelleted, and re-suspended in serum-free media. Cells were seeded into transparent transwells with 8 μm pore size (Corning, Corning, NY) mounted in 24 well plates. Serum-containing media was added in the bottom of the well below the transwells, and cells were incubated in normoxia for 15 h at 37°C. Migration was assessed by fixing cells in methanol and staining with crystal violet (Sigma). Photos of five random fields in the transwells were captured and cells were counted for analysis. Data are presented as proportion of migrated cells from total cell count.

### GSH and NADPH Assay

Cells were seeded into white-walled, clear-bottom 96-well plates (Corning) and grown overnight in normoxia. The next day, plates were transferred to hypoxia or maintained in normoxia for 48 h. Following incubation, cells were washed once with PBS, and analyzed for GSH/GSSG or NADPH/NADP^+^ ratios using GSH/GSSG-Glo Assay or NADPH/NADP^+^ Glo Assay kits (Promega) according to the manufacturer’s instructions.

### ROS Assay

Intracellular ROS was measured with 2’,7’-dichlorofluorescein diacetate (DCFH-DA) as described previously (29, 30). Cells were seeded into black-walled 96-well plates and grown overnight in normoxia. Plates were then incubated for 48 h in normoxia or hypoxia. After incubation, media was removed and cells were washed once with PBS. Cells were incubated in 10 μM DCFH-DA in PBS for 20 minutes prior to measuring fluorescence in a Synergy H1 Multi-Mode reader (excitation/emission 485/530 nm). Cell viability was determined using the MTT assay immediately following fluorescence measurement and used to normalize fluorescence.

### Metabolic Tracing Analysis

Cells were seeded into 60-mm dishes and grown in normoxia overnight. The following day, media was changed and dishes were either maintained in normoxia or transferred to a two-port zipper-lock AtmosBag glove bag (gas volume 50 L, Sigma). The glove bag was flushed with at least 750 L of 1% oxygen gas, sealed, and incubated at 37°C. All dishes were incubated for 46 h. After incubation, media on all dishes was replaced with fresh media containing 5 mM glucose and 2 mM glutamine with either 100% universally labeled ^13^C_6_ glucose or 100% universally labeled ^13^C_5_ glutamine for 2 hours at 37°C. For hypoxic samples, media was changed inside the glove bag to maintain hypoxia for the duration of the experiment. Dishes were then removed from normoxia or the hypoxic glove bag and rapidly scraped into a 70% ethanol solution heated to 70°C (31). Cells were vortexed, heated at 95°C for 5 minutes, and chilled on ice for 5 minutes. Cell debris was pelleted by centrifugation at 18,000 x g for 5 minutes. Supernatants were dried under a stream of nitrogen and the dried samples were derivatized with methoxylamine hydrochloride in pyridine and prepared with *N-tert*-butyldimethylsilyl-*N*-methyltrifluoroacetamide with 1% (wt/wt) *tert*-butyldimethylchlorosilane. Samples were analyzed with gas chromatography mass spectrometry (Thermo TSQ 8000 triple quadrupole mass spectrometer coupled with a Thermo Trace 1310 gas chromatrophagy). Metabolic ^13^C tracing was calculated by determining the percentage of labeled metabolites compared to total metabolite pool following IsoCor correction.

### siRNA Transfection

Cells were transfected with ON-TARGETplus SMARTpool siRNA against murine ATF4 (siATF4) or nontargeting control (siCtrl, Dharmacon, Lafayette, CO) using polyethylenimine (Polysciences, Warrington, PA) in serum-free media. After 12 h, transfection media was replaced with fresh serum-containing media, and cells were grown for an additional 48 h in hypoxia or normoxia prior to analysis.

### ATF4 Overexpression

Mouse ATF4 (CHOP11/cATF)-WT was a gift from David Ron (Addgene plasmid # 21845). Cells were transfected with ATF4 overexpression vector (pcDNA3.1-Atf4) or an empty vector control (pcDNA3.1-EV, ThermoFisher) using lipofectamine 2000 (ThermoFisher) according to the manufacturer’s protocol. Cells were cultured in normoxia for 48 h prior to analysis.

### Flow Cytometry

Cells were treated as indicated in figures and figure legends. Dead cells were quantified using Zombie NIR (Biolegend #423105). Spent media and trypsinized cells were centrifuged at 300 x g for 5 minutes and washed with PBS fortified with 2% BSA and 2 mM EDTA. Cells were then incubated with Zombie NIR (1:600) for 15 minutes in the dark prior to analysis in a CytoFLEX flow cytometer (Beckman Coulter). Cells were sorted by forward scatter height and side scatter height to remove debris, and cell counts (y) were plotted against APC-A (x) for quantification.

### Statistical Analysis

Kaplan-Meier (KM) plots for overall survival and relapse-free survival were generated using the KM Plotter online tool (kmplot.com), which calculates a log-rank *P* value. All cohorts/datasets were used for KM analysis, and analysis was not restricted by molecular subtype, lymph node status, grade, TP53 status, or treatment status. A total of 1879 patients were included in analysis. All values in bar graphs are presented as mean + SEM. All statistics were analyzed using SAS software version 9.4. P values < 0.05 were considered significant.

## Results

### Hypoxia Differentially Modifies Glutamine Metabolism in Nonmetastatic and Metastatic Cells

**Figure 1:**
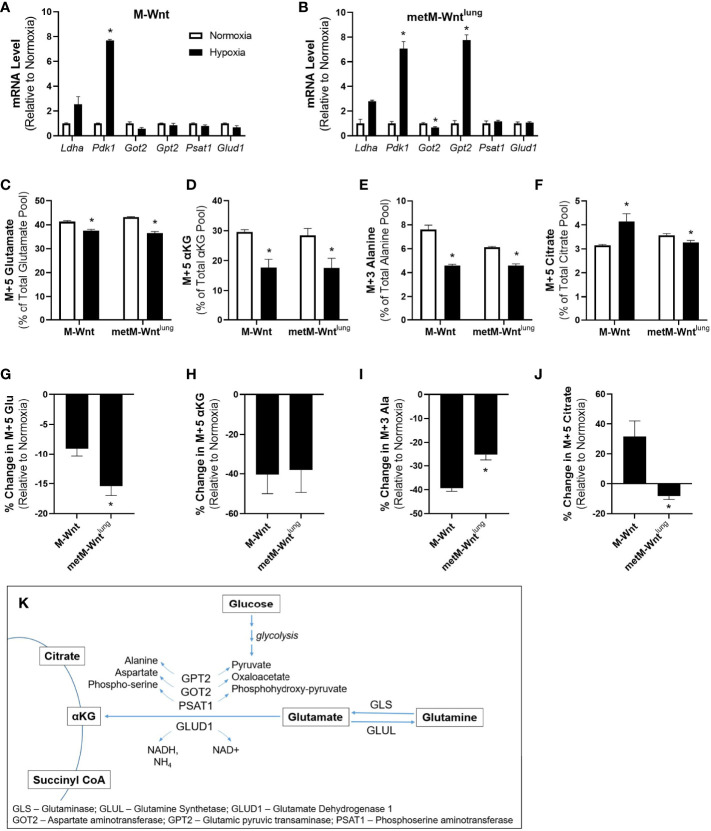
Metabolic adaptation in hypoxia. Levels of genes involved in glucose and glutamine metabolism were analyzed by qRT-PCR in M-Wnt **(A)** and metM-Wnt^lung^ **(B)** cells. Stably labeled ^13^C_5_ glutamine was used to determine metabolism to glutamate **(C)**, α-ketoglutarate **(D)**, and citrate **(F)**. Percent change in labeling of glutamate **(G)**, α-ketoglutarate **(H)**, and citrate **(J)** were determined relative to normoxia for each cell line. Stably labeled ^13^C_6_ glucose was used to determine metabolism of glucose to alanine **(E)**. Percent decrease in alanine labeling **(I)** was determined relative to normoxia for each cell line. Overview of glutamine and glucose metabolism **(K)**. Results are expressed as mean + SEM. Asterisk (*) indicates *P* < 0.05 relative to normoxia **(A–F)** or relative to M-Wnt **(G–J)**; n=3-4.

Metabolic reprogramming is a cellular adaptation employed in hypoxia to minimize the stress of reduced oxygen tension (6). Expression of genes related to glucose and glutamine metabolism, and metabolic flux were assessed to determine the effect of hypoxic incubation on metabolic reprogramming in nonmetastatic M-Wnt and metastatic metM-Wnt^lung^ cells. Hypoxia increased mRNA levels of *Pdk1*, an enzyme involved in blocking flow of pyruvate into the TCA cycle, in both M-Wnt (**Figure 1A**) and metM-Wnt^lung^ cells (**Figure 1B**). Hypoxia did not affect expression of genes related to glutamine catabolism in M-Wnt cells (**Figure 1A**), but decreased mRNA levels of the transaminase glutamic oxaloacetic transaminase 2 (*Got2*) by 33% in metM-Wnt^lung^ cells (**Figure 1B**). In contrast, hypoxia increased mRNA levels of the transaminase glutamic pyruvic transaminase (*Gpt2*) 7.7-fold in metM-Wnt^lung^ cells (**Figure 1B**). GPT2 protein levels were unchanged by hypoxia in either cell line (data not shown), a finding that is consistent with the results of glutamine metabolic tracing experiments, and that suggests post-transcriptional mechanisms contribute to *Gpt2* expression in hypoxic metM-Wnt^lung^ cells. Overall, these data indicate that hypoxia reprograms glutamine metabolism to different extents in metastatic compared to nonmetastatic cells, and that hypoxia elicits a transcriptional response in metastatic cells that is not present in nonmetastatic cells.

Given the differences in the effect of hypoxia on gene expression between the M-Wnt and the metM-Wnt^lung^ cells, ^13^C_5_ labeled glutamine or ^13^C_6_ labeled glucose were used to perform metabolite tracing of these key nutrients. Hypoxia reduced conversion of labeled glutamine to M+5 glutamate in both cell lines (**Figure 1C**), but to a greater extent in metM-Wnt^lung^ cells (13%) compared to M-Wnt cells (7%) (**Figure 1G**). In addition, hypoxia reduced conversion of labeled glutamine to M+5 αKG to similar extents in M-Wnt cells (39%) and metM-Wnt^lung^ cells (37%) (**Figures 1D, H**). Finally, hypoxia reduced conversion of labeled glucose to M+3 alanine, a product of the GPT2 reaction, in both cell lines (**Figure 1E**), and this inhibition occurred to a greater extent in M-Wnt cells (46%) compared to metM-Wnt^lung^ cells (35%) (**Figure 1I**). Taken together, these results suggest that hypoxia suppresses glutamine and glutamate catabolism in both cell lines with a greater effect in the M-Wnt cells, despite transcriptional upregulation of *Gpt2* in metM-Wnt^lung^ cells.

Reductive carboxylation of αKG to citrate has been observed in hypoxic cells (13) and was thus assessed through measurement of glutamine-derived M+5 citrate to determine the flow of carbon through the reverse TCA cycle. Metabolomic analysis of M-Wnt cells showed increased labeling of M+5 citrate from glutamine in hypoxia compared to normoxia, suggesting that M-Wnt cells display increased reductive carboxylation of αKG in hypoxia (**Figure 1F**). The reductive carboxylation reaction is mediated by isocitrate dehydrogenase-1 (cytosolic) or -2 (mitochondrial) (32). Both reactions consume NADPH and produce NADP^+^, which may increase oxidative stress in M-Wnt cells. In contrast, in metM-Wnt^lung^ cells, the pool of glutamine-derived M+5 citrate was reduced in hypoxia compared to normoxia (**Figure 1F**), suggesting that hypoxia downregulates reductive carboxylation in this cell line, potentially sparing NADPH consumption. In sum, these data indicate that hypoxia differentially regulates metabolism of glutamine-derived αKG in nonmetastatic and metastatic cells, and may suggest that hypoxia reprograms metabolism to reduce oxidative stress in metastatic cells.

### Hypoxia Induces an Oxidative Stress Response Signature in Metastatic Cells

**Figure 2:**
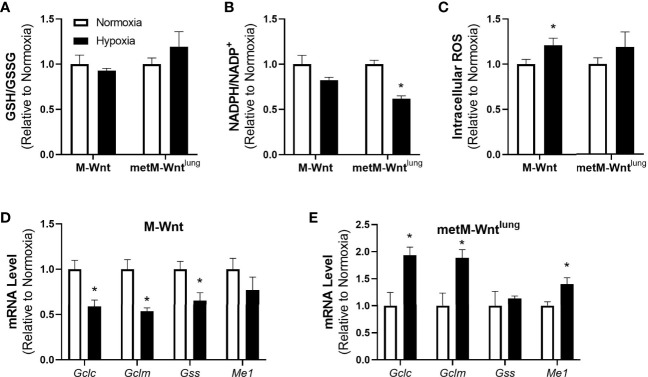
Effect of hypoxia on oxidative stress markers. Ratios of GSH/GSSG **(A)**, NADPH/NADP^+^ **(B)**, and levels of intracellular ROS **(C)** were determined. Levels of genes involved in antioxidant defense were assessed by qRT-PCR in normoxic and hypoxic M-Wnt **(D)** and metM-Wnt^lung^ **(E)** cells. Results are expressed as mean + SEM. Asterisk (*) indicates *P* < 0.05 relative to normoxia, n=3-6.

Because the metabolic tracing studies suggest a potential difference in oxidative stress between the M-Wnt and the metM-Wnt^lung^ cells, oxidative stress response, the effect of hypoxia on reductive-oxidative balance and antioxidant defense was assessed. The effect of hypoxia on overall reductive-oxidative balance was determined by assessing the ratio of reduced-to-oxidized glutathione (GSH/GSSG) and NADPH/NADP^+^. Hypoxia did not affect GSH/GSSG or GSH levels in either cell line (**Figure 2A**, GSH data not shown), but significantly reduced the NADPH/NADP^+^ ratio by 38% in metM-Wnt^lung^ cells (**Figure 2B**). In addition, hypoxia increased intracellular ROS by 21% in M-Wnt cells (**Figure 2C**). To determine if hypoxic incubation induced an antioxidant response, mRNA levels of genes involved in synthesis of glutathione and NADPH regeneration were measured. Hypoxia increased mRNA level of genes required for *de novo* glutathione synthesis including glutamate cysteine ligase-catalytic subunit (*Gclc*) and –modifer subunit (*Gclm*) by 1.9-fold in metM-Wnt^lung^ cells (**Figure 2E**). Hypoxia also significantly increased mRNA level of malic enzyme 1 (*Me1*), an enzyme which produces the antioxidant NADPH from NADP^+^, in metM-Wnt^lung^ cells (**Figure 2E**). Conversely, culturing M-Wnt cells in hypoxia suppressed mRNA levels of *Gclc*, *Gclm*, and glutathione synthetase (*Gss*) (**Figure 2D**). These data suggest that hypoxia may increase oxidative stress in metastatic metM-Wnt^lung^ cells, but not M-Wnt cells, and that the increase in oxidative stress in metastatic cells is countered by increased antioxidant response.

### Hypoxia Induces ATF4 in Metastatic Cells

**Figure 3:**
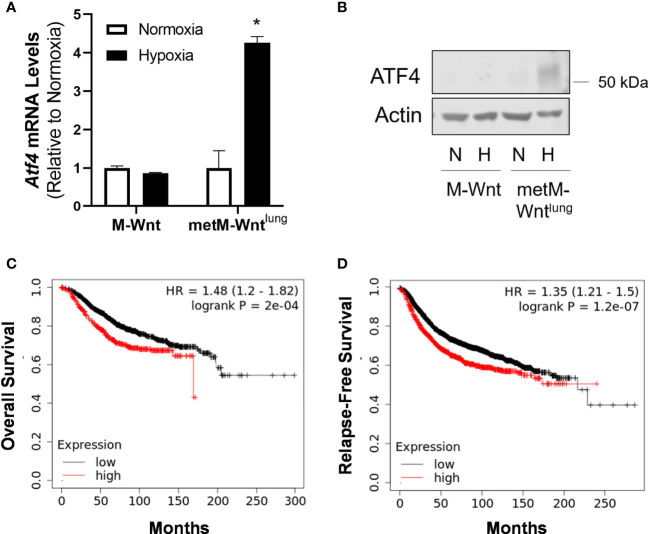
Effect of hypoxia on ATF4 expression. mRNA **(A)** and protein **(B)** expression of ATF4 was determined using qRT-PCR and Western blotting in M-Wnt and metM-Wnt^lung^ cells incubated in hypoxia (H) or normoxia (N) for 48 h. Results are expressed as mean + SEM. Asterisk (*) indicates *P* < 0.05 relative to normoxia, n=3. Overall survival **(C)** and relapse-free survival **(D)** of breast cancer patients with high (red) or low (black) expression of ATF4, where patient data are split by upper quartile (compared to lowest three quartiles) of ATF4 expression.

The ISR is activated in response to several stress stimuli, including hypoxic and oxidative stresses (9). Because metM-Wnt^lung^ cells display increased oxidative stress and upregulation of the ATF4 target gene *Gpt2* in hypoxia, ATF4 expression was evaluated in normoxic and hypoxic conditions (21, 33). Hypoxia increased ATF4 mRNA levels and protein expression in metM-Wnt^lung^ cells (**Figures 3A, B**). In contrast, hypoxia did not affect ATF4 expression in M-Wnt cells (**Figures 3A, B**), suggesting a differential response between the cell lines.

The association between ATF4 levels and survival in patients with breast cancer was assessed using KMplotter. High levels of ATF4 in patients with breast cancer were associated with poorer overall survival and relapse-free survival (**Figure 3C, D**) when patient data were split by upper quartile of expression compared to the lower three quartiles, as well as when patient data were split by median expression (data not shown).

### ATF4 Contributes to Survival of Hypoxic Metastatic Cells in Detached Conditions

**Figure 4:**
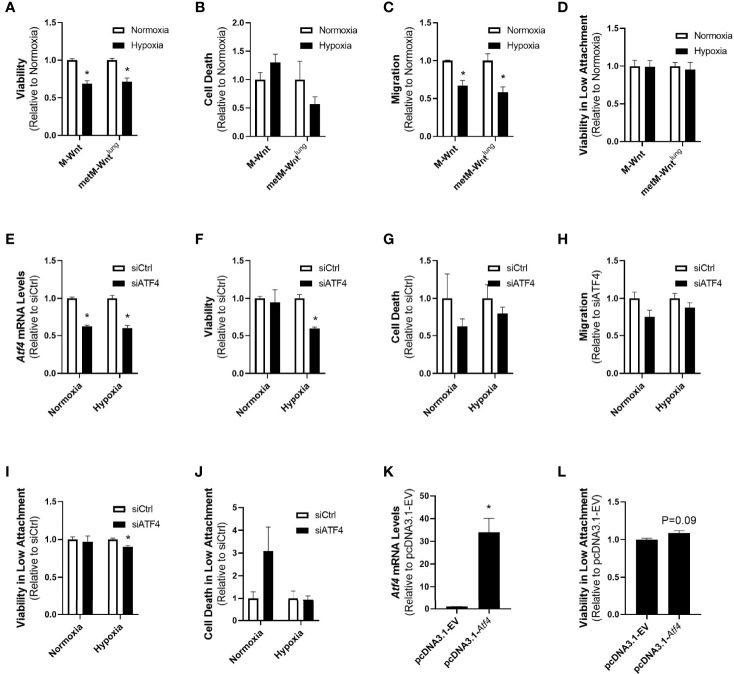
Effect of ATF4 manipulation in normoxic and hypoxic cells. Viability after 48 h in hypoxia or normoxia was assessed by MTT **(A)**. Cells were pre-incubated in normoxia or hypoxia for 48 h and cell death was determined by flow cytometry with Zombie NIR **(B)**, re-plated into transwells to assess migration **(C)** or low attachment plates to assess viability in detached conditions **(D)**. metM-Wnt^lung^ cells were transfected with siCtrl or siATF4 and incubated in hypoxia or normoxia for 48 h. ATF4 depletion was confirmed by qRT-PCR **(E)**. Cell viability **(F)** and cell death **(G)** were assessed at the end of incubation. Cells pre-incubated in hypoxia or normoxia were seeded into transwells to assess migration **(H)** or low attachment plates to assess viability **(I)** and cell death **(J)** in detached conditions. M-Wnt cells were transfected with pcDNA3.1-EV or pcDNA3.1-*Atf4* and incubated in normoxia for 48 h. ATF4 overexpression was confirmed by qRT-PCR **(K)**. Cells were seeded into low attachment plates to assess viability in detached conditions **(L)**. Results are expressed as mean + SEM. Asterisk (*) indicates *P* < 0.05 relative to normoxia **(A–D)** or *P* < 0.05 relative to siCtrl or empty vector **(E–K)**, n=3-6.

Due to the differences in expression of ATF4 between nonmetastatic and metastatic cells, and because ATF4 is reported to play a role in mediating several processes involved in metastatic progression, we compared the effects of hypoxic incubation on cell viability, migration, and viability in low attachment between the two cell lines. Hypoxia reduced viability of both M-Wnt cells (31% reduction) and metM-Wnt^lung^ cells (29% reduction) (**Figure 4A**), but did not increase cell death as measured by Zombie NIR (**Figure 4B**). Pre-incubating M-Wnt and metM-Wnt^lung^ cells in hypoxic conditions significantly suppressed migration by 33% and 42%, respectively (**Figure 4C**). In contrast, hypoxia had no significant effect on viability in detached conditions for either cell line (**Figure 4D**). These data collectively suggest that hypoxia does not differentially affect viability or processes involved in metastasis in cells with different metastatic potential, despite differences in ATF4 expression in hypoxic metM-Wnt^lung^ cells.

While both cell lines displayed similar responses to hypoxic incubation despite the high levels of ATF4 in hypoxic metM-Wnt^lung^ cells, it is unclear whether elevated ATF4 levels are required for metastatic cells. To specifically test the role of ATF4 in metastatic processes in hypoxic cells, siRNA was used to genetically deplete ATF4 in metM-Wnt^lung^ cells, and cell viability, migration, and viability in low attachment conditions were measured. Depletion of ATF4 was confirmed with qRT-PCR (**Figure 4E**). Genetic depletion of ATF4 prior to hypoxic incubation significantly reduced viability of metM-Wnt^lung^ cells, but had no effect on viability in normoxia (**Figure 4F**), and did not induce cell death at either oxygen level (**Figure 4G**). ATF4 depletion had no effect on migration in metM-Wnt^lung^ cells cultured in normoxia or hypoxia (**Figure 4H**). In contrast, metM-Wnt^lung^ cells with ATF4 depletion that were cultured in hypoxia demonstrated a 10% decrease in viability in detached conditions compared to hypoxic ATF4-expressing cells (**Figure 4I**). However, ATF4 depletion had no effect on viability in detached conditions in normoxic metM-Wnt^lung^ cells, nor did depletion increase cell death in detached cells either oxygen condition (**Figure 4J**). These results suggest hypoxic metM-Wnt^lung^ cells utilize ATF4 to maintain viability in low attachment, whereas hypoxic M-Wnt cells rely on an ATF4-independent mechanism to facilitate their viability in detached conditions following hypoxic incubation.

In order to test the hypothesis that ATF4 expression is sufficient to increase viability of cells in detached conditions, normoxic M-Wnt cells, which express low levels of ATF4, were transfected with an *Atf4* overexpression construct prior to seeding in low attachment plates. *Atf4* overexpression increased *Atf4* mRNA levels compared to control-transfected cells (**Figure 4K**) and showed a trend towards increasing survival of detached non-metastatic cells (**Figure 4L**, 8%, *P*=0.09). These results suggest ATF4 alone supports a trend towards survival in normoxic non-metastatic cells, and ATF4 plays a role in survival of metastatic cells in detachment.

## Discussion

Adaptation to cell stress is a requirement for progression of cancer to metastatic disease, and may therefore serve as a target for prevention of metastasis (34). The current study demonstrates that hypoxia drives differential responses to hypoxic stress in nonmetastatic compared to metastatic murine mammary cancer cells. Hypoxia suppressed glutamine catabolism in both cell lines, increased mRNA levels of genes associated with antioxidant defense, and had a greater effect in suppressing glutamine catabolism in metastatic metM-Wnt^lung^ cells (**Figures 1** and **2**). Furthermore, hypoxia stimulated expression of the ISR effector protein ATF4, which contributed to viability of hypoxic metM-Wnt^lung^ cells in low attachment (**Figures 3** and **4**). These findings highlight ATF4 as a potential therapeutic target to specifically inhibit outgrowth of metastatic cells in cancer patients, thus improving patient survival.

Hypoxia is a state of high oxidative stress, and continued cell survival in hypoxic conditions requires activation of antioxidant response systems to neutralize ROS (7). For example, hypoxia-inducible factor 1α directly transactivates enzymes, including phosphoglycerate dehydrogenase, which produce the reducing equivalent NADPH as byproducts of their reactions (6). In addition, cancer cells incubated in hypoxia display activation of the nuclear factor erythroid 2-related factor 2 (NRF2) signaling pathway, which promotes transcriptional activation of genes whose protein products reduce ROS and oxidative stress, including *Gclc*, *Gclm*, and *Me1* (7, 35–38). Both GCLC and GCLM contribute to *de novo* synthesis of GSH, which can eliminate ROS (e.g. hydrogen peroxide) in the glutathione peroxidase reaction *via* its oxidation to GSSG (7). ME1 mediates the conversion of NADP^+^ to NADPH, thus providing NADPH which recycles GSSG to GSH, enabling further clearance of ROS by glutathione.

Our data show that only metastatic metM-Wnt^lung^ cells increased mRNA levels of genes related to *de novo* synthesis of the antioxidant glutathione and cellular regeneration of NADPH in response to hypoxia (**Figure 2**). This observation suggests that hypoxia increases oxidative stress such that NRF2 signaling is activated in metastatic cells compared to nonmetastatic cells, and is consistent with results from previous literature that show increasing levels of ROS and ROS-related cell damage throughout the course of metastatic progression (39). Additionally, in metastatic metM-Wnt^lung^ cells the NADPH/NADP^+^ ratio was significantly lower in hypoxia compared to normoxia, whereas the GSH/GSSG ratio and ROS levels were unaffected by hypoxia (**Figure 2**). These data may suggest that NADPH was utilized for recycling GSSG to GSH for clearance of ROS, although further evaluation is required to test this hypothesis. In M-Wnt cells, hypoxia significantly increased intracellular ROS accumulation but did not affect NADPH/NADP^+^ or GSH levels or GSH/GSSG ratios or increase transcriptional activation of genes related to antioxidant defense. Given that the genes encoding glutathione synthesis enzymes are reduced in hypoxic M-Wnt cells, but glutathione levels are not altered, the small but significant increase in ROS may not be sufficient to require or stimulate increased production of glutathione and would thus not alter the GSH/GSSG ratio. Further investigation is required to determine the mechanism involved in reducing the expression of *Gclc*, *Gclm* and *Gss* in the M-Wnt cell line. Collectively, our results suggest that metastatic cells activate antioxidant programs in response to hypoxia to alleviate oxidative stress.

Another mechanism by which hypoxic cells reduce oxidative stress is by limiting production of ROS *via* metabolic reprogramming. Metabolic reprogramming in hypoxic cells favors metabolism of glucose through glycolysis and limits oxidation of glucose, fatty acids, and glutamine through the forward TCA cycle. For example, previous literature has shown that hypoxia inhibits oxidation of glutamine-derived αKG, and instead promotes reductive carboxylation of αKG to citrate for fatty acid synthesis (13). In the present study, metabolic reprogramming of glutamine metabolism in hypoxia varied between nonmetastatic and metastatic cells (**Figure 1**). While hypoxia suppressed conversion of glutamine to glutamate in both cell lines, the percent decrease in M+5 labeled glutamate in hypoxia was significantly higher in metM-Wnt^lung^ cells compared to M-Wnt cells (**Figure 1**), suggesting that hypoxia suppresses glutaminolysis to a greater extent in metastatic cells. These results are striking, as previous research showed that cancer cells have increased glutamine catabolism and glutaminase activity throughout the course of progression (40). Furthermore, glutaminase is induced by hypoxia-inducible factor 1α in colon cancer cells and plays a pro-metastatic role in colon cancer metastasis (41). Together, these observations suggest that regulation of glutamine metabolism and glutamine dependence under hypoxic conditions varies between different cancer types. In addition, the present work shows that hypoxia increases reductive carboxylation of glutamine-derived αKG to citrate in nonmetastatic cells but decreases this pathway in metastatic cells (**Figure 1**). These data may suggest that hypoxia differentially regulates activity of isocitrate dehydrogenase, the enzyme responsible for conversion of αKG to isocitrate, or the αKG-dehydrogenase inhibitor Siah E3 Ubiquitin Protein Ligase 2 (SIAH2) in nonmetastatic compared to metastatic cells. Previous literature has identified hypoxia as a regulator of both isocitrate dehydrogenase and SIAH2 in several types of cancer (6), although this work has not been studied specifically in models of cancer progression.

We also demonstrate that hypoxia significantly increased the mRNA level of the transaminase *Gpt2* only in metM-Wnt^lung^ cells (**Figure 1**). Previous literature shows that ATF4, the main effector protein of the ISR pathway, induces *Gpt2* mRNA levels (21). The results of the present study show that high ATF4 expression was associated with poorer overall survival and relapse-free survival in breast cancer patients (**Figure 3**), that ATF4 was activated in hypoxic metM-Wnt^lung^ cells, and contributed to viability of hypoxic metM-Wnt^lung^ cells in adherent and detached conditions (**Figure 4**). We observed that ATF4 depletion more strongly suppressed viability of cells in adherent conditions compared to detached conditions, suggesting that ATF4 mediates continued proliferation of attached cells in addition to its role in promoting viability in detachment. In addition, depletion of ATF4 may modestly increase cell death in normoxic cells (**Figure 4**), suggesting that low levels of ATF4 are required for cell survival in unstressed conditions. Our results are in agreement with previous research which shows that ATF4 induction in cancer cells contributes significantly to anoikis resistance in fibrosarcoma cells (20). However, ATF4 was also identified as a driver of cell migration in a model of breast cancer (18), whereas ATF4 depletion had no effect on migration in the present study. ATF4 mediated its effects on cell migration through upregulation of the zinc finger E-box binding homeobox 1, a transcription factor that regulates the epithelial-to-mesenchymal transition, suppresses E-cadherin, and increases migration (18). While metM-Wnt^lung^ cells have undergone a partial mesenchymal-to-epithelial transition compared to mesenchymal M-Wnt cells, both cell lines express low levels of E-cadherin and high levels of the mesenchymal markers vimentin and snail (27). These results may suggest that both cell lines are sufficiently mesenchymal to drive migration independent of ATF4 activation, and may explain why ATF4 did not affect cell migration in the present study.

The results of this study suggest that hypoxic incubation activates the NRF2 pathway in metM-Wnt^lung^ cells. First, our data show increased transcriptional activation of classic NRF2 target genes involved in antioxidant response only in hypoxic metM-Wnt^lung^ cells (38). In addition, our results show transcriptional activation of *Atf4* in hypoxic metM-Wnt^lung^ cells (**Figure 3**). These results are of particular interest, as previous research showed that hypoxia only induced translational activation of ATF4, whereas transcriptional activation of *Atf4* can be mediated by NRF2 signalling (8, 15). Thus, our results collectively suggest that hypoxia induces *Atf4* through NRF2 activation. A limitation of the present work is that NRF2 pathway activity was not measured to assess this hypothesis. Because previous literature suggests that extracellular matrix detachment increases oxidative stress in cancer cells (42–44), hypoxia-mediated induction of NRF2 and *Atf4* may play a critical role in priming metastatic cells to survive in high stress conditions, including during matrix detachment. In addition to the NRF2 pathway, other regulators of ATF4 expression, including the integrated stress response protein PKR-like ER kinase, may also contribute to increased ATF4 expression in hypoxic metM-Wnt^lung^ cells.

Collectively, our results show that nonmetastatic and metastatic murine mammary cancer cells utilize different mechanisms to facilitate their survival in stress conditions. Hypoxia drives expression of ATF4, increases mRNA levels of antioxidant defense genes and facilitates suppression of glutamine catabolism in metastatic metM-Wnt^lung^ cells to a greater extent relative to nonmetastatic M-Wnt cells. These results warrant further investigation to address the interaction between antioxidant response and ATF4 activation in hypoxic metastatic cells, and may help elucidate molecular targets to prevent metastatic progression in patients with cancer.

## Data Availability Statement

The raw data supporting the conclusions of this article will be made available by the authors, without undue reservation.

## Author Contributions

VK and DT both designed experiments and wrote the manuscript. VK, EH, and CA conducted experiments and performed data analysis. MS and SD contributed to metabolic tracer experiments. All authors contributed to experimental design, reviewing the content of the manuscript, and approved the final manuscript.

## Funding

This work was supported by the Purdue University Center for Cancer Research; Indiana Clinical Translational Science Institute NIH/NCRR [#TR000006], the American Cancer Society [RSG-CSM130259] and the National Institute of Health [R01CA232589, R35CA197627, R01CA207751, R01CA232589, R21AA026675].

## Conflict of Interest

The authors declare that the research was conducted in the absence of any commercial or financial relationships that could be construed as a potential conflict of interest.

## Publisher’s Note

All claims expressed in this article are solely those of the authors and do not necessarily represent those of their affiliated organizations, or those of the publisher, the editors and the reviewers. Any product that may be evaluated in this article, or claim that may be made by its manufacturer, is not guaranteed or endorsed by the publisher.

## Abbreviations

αKG, α-ketoglutarate; ATF4, activating transcription factor 4; GPT2, glutamic pyruvic transaminase 2; GCLC, glutamate cysteine ligase catalytic subunit; GCLM, glutamate cysteine ligase modifier subunit; GLUD1, glutamate dehydrogenase 1; GOT2, glutamic oxaloacetic transaminase 2; GSH, reduced glutathione; GSS, glutathione synthetase; GSSG, oxidized glutathione; ISR, integrated stress response; LDHA, lactate dehydrogenase A; ME1, malic enzyme 1; NADPH, nicotinamide adenine dinucleotide phosphate; NRF2, nuclear factor erythroid 2-related factor 2; PDK1, pyruvate dehydrogenase kinase 1; PSAT1, phosphoserine aminotransferase; ROS, reactive oxygen species; TCA, tricarboxylic acid cycle.
